# The role of β-adrenergic receptor signaling in the proliferation of hemangioma-derived endothelial cells

**DOI:** 10.1186/1747-1028-8-1

**Published:** 2013-01-03

**Authors:** Yi Ji, Siyuan Chen, Kai Li, Xianmin Xiao, Shan Zheng, Ting Xu

**Affiliations:** 1 Division of Oncology, Department of Pediatric Surgery, Children’s Hospital of Fudan University, 399 Wanyuan Road, Shanghai, 201102, China; 2 Research Institute of Pediatrics, Children’s Hospital of Fudan University, Shanghai, 201102, China

**Keywords:** Infantile hemangioma, Endothelial cells, Cell cycle, Proliferation

## Abstract

**Background:**

Infantile hemangioma (IH) is a benign vascular neoplasm that arises from the abnormal proliferation of endothelial cells and enhanced angiogenesis. Recently, propranolol has been found to be effective in the management of IH, suggesting that β-adrenergic receptors (β-ARs) may play an important role in the pathogenesis of IH.

**Results:**

In the present study, we investigated the β-adrenergic signaling that is associated with hemangioma-derived endothelial cell (HemEC) proliferation. The results showed that both β_1_- and β_2_-ARs were expressed in HemECs. Stimulation of the β-ARs by isoprenaline induced cell proliferation and elevation of second messenger cAMP levels. The proliferation-promoting action of isoprenaline was abolished by a β_1_-selective antagonist and was more effectively abolished by a β_2_-selective antagonist; the mechanism for the action of the antagonists was a G_0_/G_1_ phase cell cycle arrest which was associated with decreased cyclin D1, CDK-4, CDK-6 and phospho-Rb expression. Pre-treatment of the cells with VEGFR-2 or ERK inhibitors also prevented the isoprenaline-mediated proliferation of cells. In agreement with the involvement of β-ARs and VEGFR-2 in the HemEC response, β-AR antagonists and the VEGFR-2 inhibitor significantly attenuated isoprenaline-induced ERK phosphorylation. Moreover, treating the cells with isoprenaline markedly increased VEGF-A expression and VEGFR-2 activity in a β_2_-AR-dependent manner.

**Conclusions:**

We have demonstrated that the activation of the β-ARs in the ERK pathway may be important mechanisms in promoting HemEC growth. Furthermore, stimulation of the β-AR may transactivate VEGFR-2 signaling and further increase HemEC proliferation.

## Introduction

Infantile hemangioma (IH) is the most common form of vascular tumor, affecting 5% to 10% of all infants and up to 30% of premature infants [1,2]. IHs occur more frequently in females than in males (at a ratio of 3:1) [3] and generally appear within the first weeks postpartum, proliferate rapidly during the first years of life, and spontaneously involute over a subsequent period of several years. The proliferating and involuting phases of IHs represent a gradual shift in the balance of the mitotic and apoptotic activities of the local endothelial cell population [4]. It has been demonstrated that vascular endothelial growth factor (VEGF) is involved in the proliferating phase of IH [5-7]. VEGF is the most potent stimulator of hemangioma-derived endothelial cell (HemEC) proliferation and differentiation [8], and the factor exhibits its pro-proliferative and pro-angiogenic functions by binding to the tyrosine kinase receptor VEGFR-2 on HemECs [9]. Many reports have confirmed that excessive VEGF expression in IH tissue parallels the proliferating phase of IH tissue growth. Conversely, during the involuting phase, VEGF expression rapidly decreases, and many inhibitors of angiogenesis are instead expressed [5-7].

For most children with IH, the lesions are small and pose no threat or potential for complication, but in some cases, IHs grow dramatically and destroy tissue, impair function, or even threaten life. Standard treatment options for IH include corticosteroids or surgical excision or, in life- or sight-threatening cases, treatment with vincristine, interferon or cyclophosphamide. Unfortunately, none of these therapeutic modalities is ideal due to restrictions or potential serious side effects [10-12]. In 2008, Léauté-Labrèze et al. [13] showed that propranolol has an anti-proliferative effect on severe IHs. After this report, a number of studies further demonstrated that β-blockers other than propranolol were effective at halting hemangioma growth with few adverse side effects [14-21]. β-blockers are now the preferred treatment for problematic proliferating IHs.

To date, it is unknown how β-blockers exert its pharmacologic effect on IHs. The β-ARs, a family of G-protein-coupled receptors that are activated by adrenergic catecholamines, can initiate a series of signaling cascades, thereby leading to multiple cell-specific responses. There is evidence suggesting that endogenous catecholamines play a role in basic developmental processes (e.g., embryogenesis and morphogenesis) including the control of cell proliferation, differentiation and migration [22-24]. Recent studies have demonstrated that in endothelial and various cancer cells, a number of β-AR agonists, including epinephrine, norepinephrine and isoprenaline, can induce the proliferation and activation of mitogen-activated protein kinase (MAPK) family members by extracellular signal-related kinase (ERK) [25-28]. ERK and MAPK are serine/threonine kinases that phosphorylate nuclear transcription factors and regulate the expression of multiple genes involved in cell proliferation. VEGF-A participates in this process because a VEGF-A-specific antibody blocks β-AR-mediated cell proliferation and ERK activation [9,25]. Additionally, VEGF-A exerts its pro-proliferative and pro-angiogenic effects, at least partially, by activating the ERK cascade [25,29]. In primary endothelial cells, VEGFR-2 also associates with activated ERK in a Ras-independent manner [30].

The mechanisms of β-AR-stimulated tumor growth have been studied for several years, but the potential role of the β-ARs in IH pathogenesis has not been investigated. Accordingly, this study examined the mechanisms underlying the relationship between the β-adrenergic signaling pathway and the proliferation of HemECs.

## Methods

### Reagents and antibodies

Endothelial basal medium (EBM-2) and SingleQuot, which contains human epidermal growth factor, vascular endothelial growth factor, human basic fibroblast growth factor, insulin-like growth factor, hydrocortisone, heparin, ascorbic acid, and gentamicin/amphotericin B, were obtained from Lonza (Walkersville, MD). Fetal bovine serum (FBS) and phosphate-buffered saline (PBS) were purchased from Gibco (Grand Island, NY). The anti-CD31 FITC antibody used in fluorescence-activated cell sorting (FACS) was obtained from BD Pharmingen (San Jose, CA). ICI 118551 (ICI), metoprolol (MET), isoprenaline (ISO), forskolin, collagenase A, bovine serum albumin (BSA), Hoechst 33342, propidium iodide, DNase-free RNase, the ERK/MAPK inhibitor, U0126, the phosphodiesterase inhibitor, 3-isobutyl-methylxanthine (IBMX), and the cAMP antagonist, Rp-cAMP, were purchased from Sigma (St. Louis, MO). The VEGFR-2 inhibitor, PTK787, was obtained from Novartis Pharmaceuticals (Basel, Switzerland). The BrdU cell proliferation assay kit was obtained from Calbiochem (Darmstadt, Germany), and the cAMP assay kit was obtained from Amersham Pharmacia Biotech (Braunschweig, Germany). The primary polyclonal antibodies recognizing the VEGF-A, β_1_-AR, β_2_-AR, phospho-ERK (Thr202/Tyr204) and ERK were purchased from Santa Cruz Biotechnology (Delaware, CA). The antibodies for cyclin D1, CDK-4, CDK-6, retinoblastoma (Rb), phospho-Rb, phospho-VEGFR-2 (Tyr1175) and VEGFR-2 were purchased from Cell Signaling Technology (Boston, MA). Human umbilical vein endothelial cells (HUVECs) were obtained from Chinese Academy of Sciences (Shanghai, China).

### Preparation of hemangioma specimens

This study was approved by the Ethics Committee of the Children’s Hospital of Fudan University. Proliferating infantile hemangioma was surgically removed from a 4-month-old female patient who was referred to our department for a rapidly growing mass. Written informed consent was obtained from parents for all tissue obtained for the study. The clinical diagnosis of vascular neoplasm was confirmed by the Department of Pathology at the Children’s Hospital of Fudan University based on staining for GLUT-1, a marker specific for hemangioma tissue. The tissues were used immediately in cell isolation and in vitro experiments.

### Cell extraction, isolation and culture

HemEC isolation was performed as described previously [7,8]. Briefly, the hemangioma samples were rinsed in PBS, minced, and digested with 0.2% collagenase A at 37°C for 1 h. The tissue was homogenized and filtered through 100 μm cell strainers to dissociate aggregates, and red blood cells were lysed by incubating the samples in NH_4_Cl. Next, the samples were filtered through a 40 μm cell strainer to obtain a single-cell suspension. CD31^+^ HemECs were isolated by FACS using anti-CD31 FITC antibodies and were plated on gelatin-coated 60 mm plates in EBM-2 medium supplemented with 20% heat-inactivated FBS, SingleQuot, penicillin (100 units/ml) and streptomycin (100 μg/ml). The cells were grown in humidified air containing 5% CO_2_ at 37°C. Cells at passage 3 to 6 were used for experiments. The purity of the HemECs was >95% as determined by positive von Willebrand factor and CD31 expression, and by negative expression of vimentin (fibroblasts) and α-actin (vascular smooth muscle cells) as previously described [8].

### Analysis of β-ARs expression

The mRNA of the β_1_- and β_2_-ARs expressed in HemECs was isolated using Trizol reagent (Invitrogen, Carlsbad, CA) and reverse transcribed into cDNA. Quantitation of the relative mRNA abundance was performed using an ABI Prism 7700 Sequence Detection System (Applied Biosystems). The glyceraldehyde-3 phosphate dehydrogenase (GAPDH) gene served as an internal control. The abundance of transcripts in the cDNA sample was measured by real-time PCR using specific primers according to the manufacturer’s instructions. The primers are listed in Table 1. The samples were performed in triplicate. For each experimental condition, at least three replicates were performed. Differences in threshold cycles between the target genes and the housekeeping gene (GAPDH) were calculated.

**Table 1:** Primers used for RT-PCR analysis

**Gene**		**Primer sequence**	**Fragment size (bp)**
β_1_-Adrenoceptor	forward	5^′^- CTCCTTCTTCTGCGAGCTGT-3^′^	204
(NM000684)	reverse	5^′^- AGGATGGGCAGGAAGGAC-3^′^	
β_2_-Adrenoceptor	forward	5^′^- ACGCAGCAAAGGGACGAG-3^′^	401
(NM000024)	reverse	5^′^- CACACCATCAGAATGATCAC-3^′^	
GAPDH	forward	5^′^-CTCAGACACCATGGGGAAGGTGA-3^′^	450
(NM001256799)	reverse	5^′^--ATGATCTTGAGGCTGTTGTCATA-3^′^	

Western blot analysis of β-AR protein expression in HemECs was performed as previously described [26]. Briefly, protein was extracted from cultured cells in radioimmunoprecipitation assay lysis buffer for 20 min on ice. The proteins were electrophoretically separated in 10% polyacrylamide gels, transferred to Hybond-ECL membranes (Amersham Bioscience), probed with either the β_1_-AR or β_2_-AR primary antibody overnight at 4°C and then probed again with secondary antibodies for 30 min. The protein bands were visualized using enhanced ECL-associated fluorography.

### Cell treatment

Before each treatment, the cells were plated and cultured in standard media as described above. After 24 h of incubation to allow for cell attachment, the cells were washed twice with PBS and synchronized by serum starvation for 24 h in EBM-2 medium containing 0.1% BSA. The medium was then removed and replaced with fresh medium containing 5% FBS. Different concentrations of ISO were added to the cells for various times to study its mitogenic effect. To examine the effects of various antagonists or inhibitors, the cells were pre-treated with the antagonists or inhibitors for 1 h before ISO treatment. SingleQuot was excluded during cell treatment.

### BrdU cell proliferation assay

A BrdU cell proliferation assay was performed according to the manufacturer’s instructions. Briefly, fresh culture medium containing BrdU (1:2000) was added, and the cells were incubated for 18 h at 37°C. After washing, 200 μl of fixative/denaturing solution was added to each well, and the cells were incubated for 30 min at room temperature. The cells were then treated with an anti-BrdU antibody (1:100) for 1 h at room temperature. For conjugation of the peroxidase goat anti-mouse IgG to the anti-BrdU antibody, 100 μl of the conjugate solution containing the secondary antibody was added to each well. Free conjugates were removed by washing with wash buffer three times and with distilled water once. After removal of the contents of the well, the reaction was stopped by adding stop solution, and the absorbances of the wells were read at 520 nm on an automatic microplate reader (Bio-Rad, CA).

### Quantification of viable cells

HemECs were plated in a 96-well plate and incubated in the absence or presence of various chemicals. The number of viable cells was determined using a CCK-8 assay kit. Briefly, 10 μl of the CCK-8 solution was added to each well, and the plate was incubated for 2 h. The absorbance of each well was measured at 450 nm using a microplate reader (Bio-Rad, CA).

### Cell cycle analysis

Cell cycle distribution was analyzed by flow cytometry (Beckman Coulter, Brea, CA). After treatment, the cells were trypsinized, centrifuged at 1,000 × g for 5 min, collected and washed with ice-cold PBS. Next, the cell pellets were resuspended and fixed with cold 70% ethanol overnight. After another wash with PBS, the cell pellets were resuspended in 1 ml of staining solution containing propidium iodide (PI, 50 μg/ml), DNase-free RNase (100 μg/ml) and Triton-100 (0.3%, Bioengineering Corporation, Shanghai, China). Finally, the cells were incubated at 37°C for 30 min in the dark before analysis. The fraction of the cell population in each phase of the cell cycle was determined as a function of the DNA content using flow cytometry analysis.

### cAMP assay

The intracellular cAMP assay was performed according to the manufacturer’s recommendations. In brief, 1 × 10^6^ cells were treated without or with ISO (1 μM) for 5 min in the presence of 100 μM IBMX. The cells were then scraped and lysed with lysis buffer (Amersham Pharmacia Biotech, Braunschweig, Germany). The levels of cAMP were measured using the enzyme immunoassay method and were expressed as picomoles of cAMP per milligram of protein.

### Western blot analysis

Western blot analysis using antibodies against cyclin D1, CDK-4, CDK-6, phospho-Rb, Rb, VEGF-A, phospho-VEGFR-2 (Tyr1175), VEGFR-2, phospho-ERK and ERK was performed on extracted proteins as previously described [9,31]. The proteins were visualized by ECL, and the intensity of the signal was quantified by scanning laser densitometry.

### Statistical analysis

All data were expressed as the mean ± SD with n = 3 for each sample for all of the paired statistical comparisons. The analysis of variance (ANOVA) test followed by Tukey’s *t*-test was performed, and a *P* value less than 0.05 was considered statistically significant.

## Results

### Expression of β-ARs in HemECs

**Figure 1:**
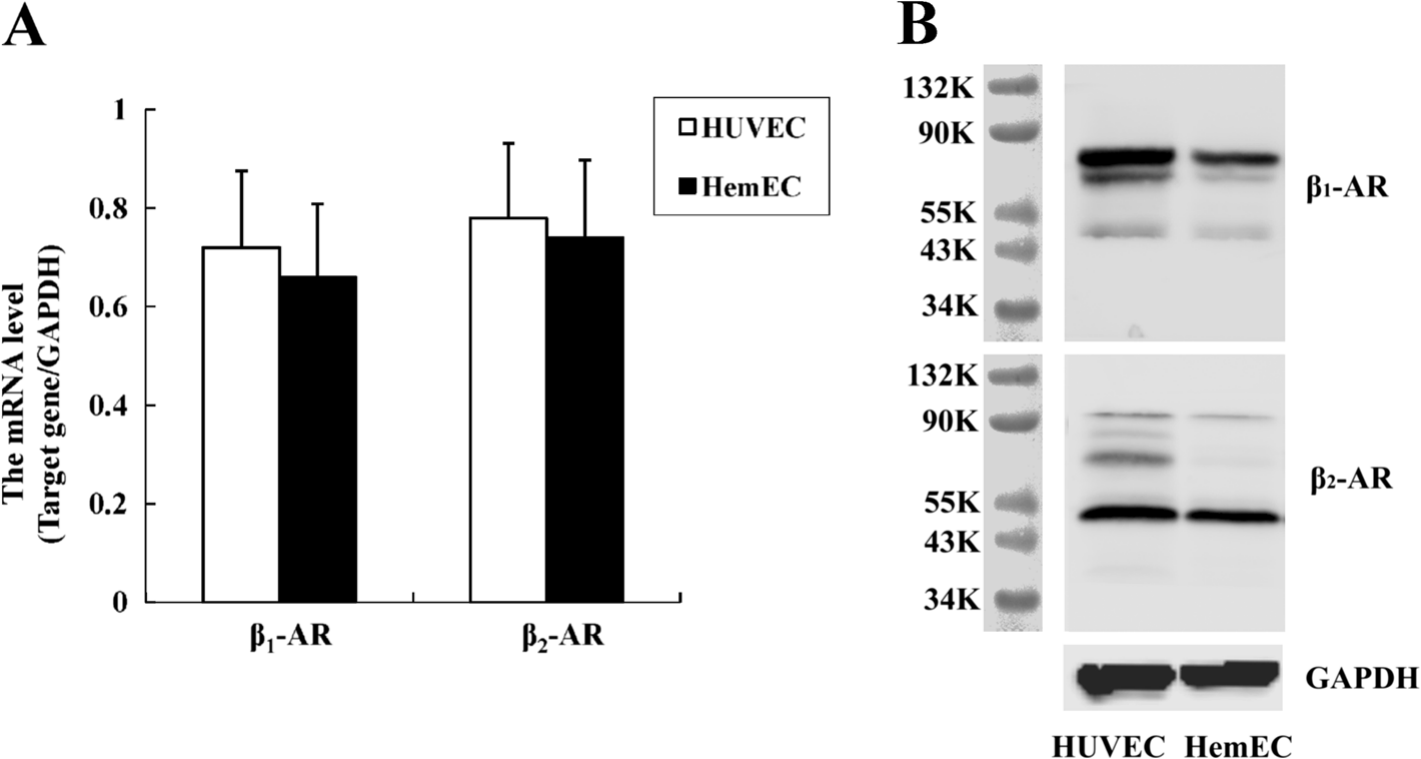
**Expression of β-ARs in HemECs. A**, Real-time PCR expression assays measure the β_1_- and β_2_-AR expression in HemECs. The data are represented as the relative abundance of each target gene normalized to the GAPDH levels. **B**, Western blot analysis of β_1_- and β_2_-AR expression in HemECs. Cell lysates probed for β_1_-AR revealed two bands with an apparent molecular weight of 65-75 kDa, and one band at 51 kDa. Two bands were observed when HemEC lysates were probed for β_2_-AR: one band with molecular weights of 47 kDa, another band at 90 kDa. These bands were not observed in blots incubated with normal rabbit serum (not shown).

Expression of the β_1_- and β_2_-ARs in HemECs was measured at the mRNA and protein levels by quantitative real-time PCR and Western blotting, respectively. HUVEC were used as control. The real-time-PCR results showed that the HemECs constitutively expressed the transcripts for both the β_1_- and β_2_-ARs (Figure 1A). Western blot analysis of β_1_- and β_2_-AR expression in the lysates of HemECs showed that these cells also expressed both of the β-ARs (Figure 1B).

### ISO increased HemECs proliferation, and the effect was reversed by β-AR antagonists

**Figure 2:**
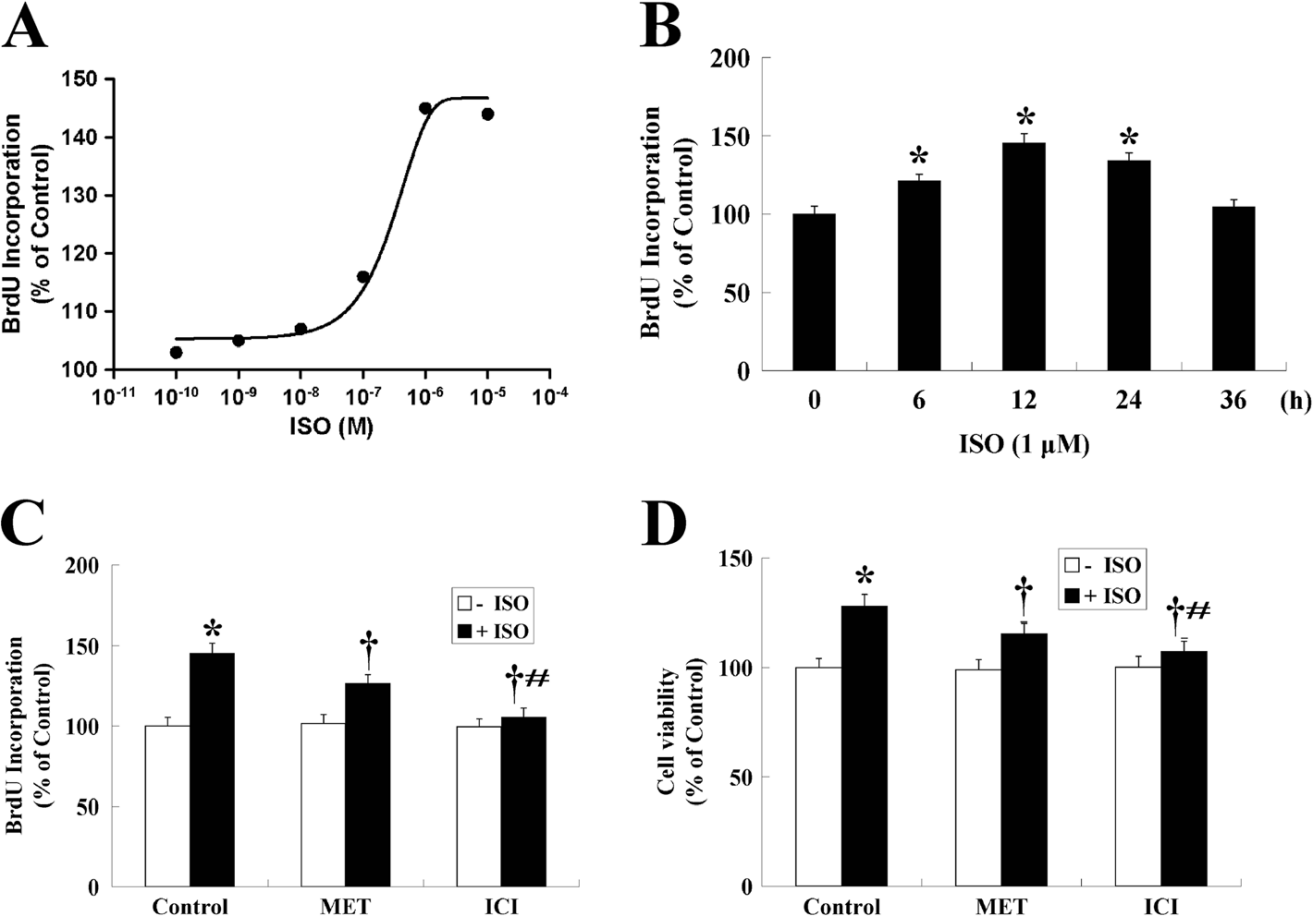
**Role of β-ARs in the proliferation of HemECs. A**, Incubation of HemECs with ISO for 12 h increased DNA synthesis in a dose-dependent manner with an EC_50_ of 340 ± 41 nM. HemECs were incubated in EBM-2 with 5% FBS and synchronized for 24 h in EBM-2 with 0.1% BSA prior to stimulation. **B**, HemECs were incubated in the presence of 1 μM ISO for various times (0-36 h). **C**, The effects of β_1_- and β_2_-AR blockade with MET and ICI on ISO-induced HemECs proliferation. HemECs were pre-treated with MET or ICI for 1 h followed by the addition of 1 μM ISO. ICI more efficiently blocked ISO-enhanced cell proliferation. **D**, The number of viable cells was counted using CCK-8. ISO treatment increased cell number, whereas MET and ICI prevented the ISO-induced increase in cell number. The results are shown as the mean ± SD of triplicate assay from one of three identical experiments. * *P*<0.05 when compared with the ISO-untreated control, ^†^*P*<0.05 when compared with the ISO-treated control, ^#^*P*<0.05 when compared with the MET-treated group.

The effect of ISO on BrdU incorporation by HemECs was examined by using various concentrations of ISO (0-10 μM) for 12 h or by treating HemECs with a fixed concentration of ISO (1 μM) for various times (0-36 h). As shown in Figure 2A and B, the level of BrdU incorporation increased at a 10 nM concentration of ISO, with a maximum stimulatory effect observed at 1 μM. Increased BrdU incorporation was first observed at 6 h; this effect peaked at 12 h and gradually decreased over a 24 h period. In addition, a significant increase in the number of cells was observed after incubation of the cells with 1 μM ISO for 12 h (Figure 2D).

The β_1_-selective antagonist, MET (10 μM; β_1_:β_2_ receptor activity, 10:1), and the β_2_-selective antagonist, ICI (10 μM; β_1_:β_2_ receptor activity, 1:100), were used to determine whether β_1_- and β_2_-ARs mediated the stimulatory action of ISO. The results showed that neither antagonist had an effect on basal cell proliferation, but both significantly decreased ISO-induced cell proliferation and cell viability. ICI was more effective than MET in reducing the ability of ISO to promote both cell proliferation and a change in cell number as showed by BrdU and CCK-8 assays, respectively (Figure 2C and D).

### The expression cell cycle regulators was upregulated by ISO but inhibited by β-AR antagonists

**Figure 3:**
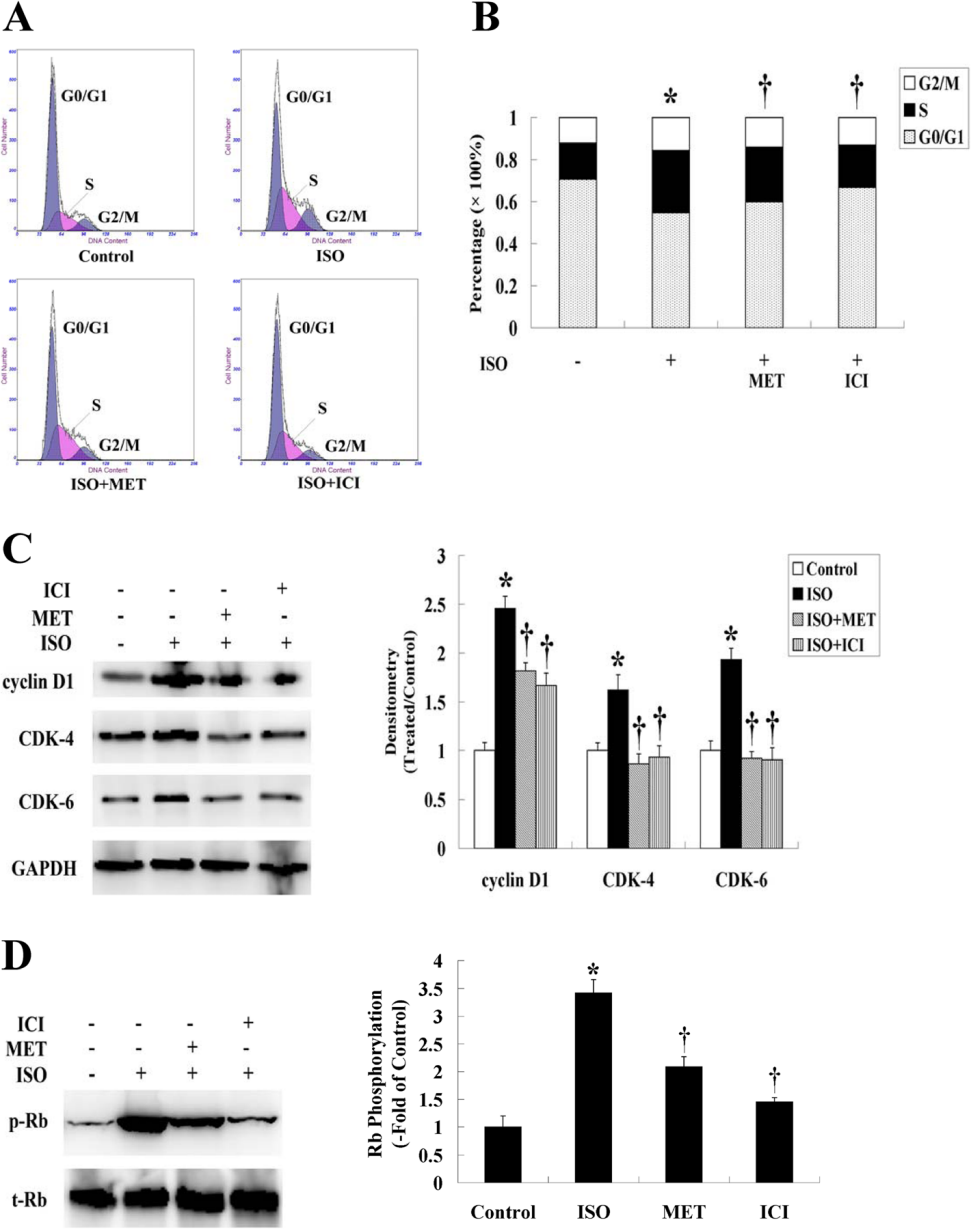
**Expression of cell cycle regulators was upregulated by ISO but abolished by β-AR antagonists. A**, Cell cycle analysis was performed to determine the different phases of the cell cycle that occurred when HemECs were treated with or without ISO, MET or ICI. The data shown were collected from 10,000 events. **B**, Histogram distribution of cells in the G_0_/G_1_, S and G_2_/M phases as determined by flow cytometry. **C**, Western blot analysis of cyclin and CDK protein expression. The expression of cyclin D1, CDK-4 and CDK-6 was upregulated by ISO but abolished by MET or ICI. GAPDH served as the loading control. **D**, Western blot analysis of Rb phosphorylation. ISO caused the phosphorylation of Rb, and this effect was inhibited by MET or ICI. Total Rb (tRb) was used for normalization. Data are representative of three independent experiments. * *P*<0.05 when compared with the ISO-untreated control, ^†^*P*<0.05 when compared with the ISO-treated control.

To investigate the mechanism responsible for β-AR stimulation of cell proliferation, we performed a cell cycle analysis in HemECs. As shown in Figure 3A and B, ISO promoted cell cycle progression from the G_1_ to S phase. Pre-treatment of HemECs with MET or ICI resulted in a greater number of cells in the G_0_/G_1_ phase and a lesser number of cells in the S phase when compared with HemECs treated with ISO alone.

Cell cycle progression is controlled by cyclins, CDKs, Rb and many other proteins. When stimulated with mitogens, dormant cells enter the cell cycle by activating cyclin D1 and its cyclin-dependent kinases, CDK-4 and CDK-6, and by phosphorylating the Rb protein to release E_2_F transcription factors [32]. To determine the level of expression of these cell cycle regulators in HemECs after ISO treatment, immunoblotting was performed. Western blot analysis confirmed that ISO not only increased the expression of cyclin D1 and its associated kinases, CDK-4 and CDK-6, but also induced the phosphorylation of Rb when compared with the control group. In contrast, pre-treatment of HemECs with β-AR antagonists significantly inhibited the stimulating effect of ISO on these regulators (Figure 3C, D).

### Cyclic AMP levels in HemECs were elevated upon ISO treatment

**Figure 4:**
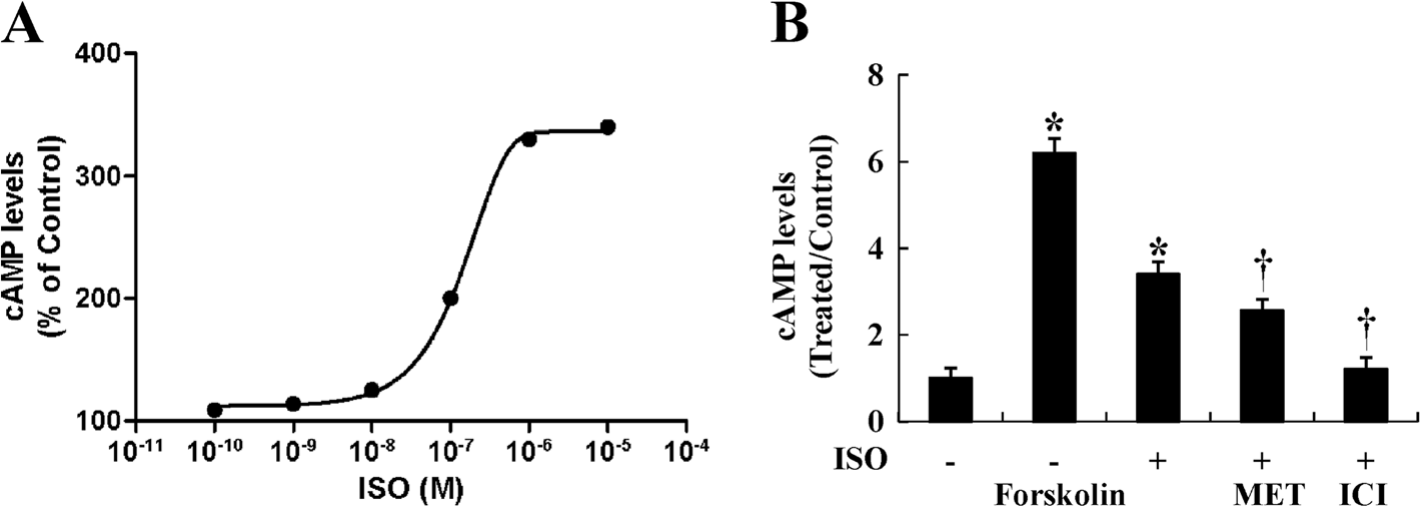
**Intracellular cAMP production was enhanced by ISO but reversed by β-AR antagonists. A**, cAMP levels after 5 min stimulation with the indicated concentrations of ISO. Curve was drawn according to simple Michaelis-Menten kinetics, yielding an EC_50_ of 180 ± 37 nM. B, HemECs were pre-treated with 10 μM MET or 10 μM ICI for 1 h followed by stimulation with 1 μM ISO for 5 min. Positive controls consisted of cells treated with only 100 μM forskolon for 5 min. All reactions took place in the presence of 100 μM IBMX. cAMP levels were measured using the enzyme immunoassay method. The results are shown as the mean ± SD of triplicate assays from one of three identical experiments. * *P*<0.05 when compared with the ISO-untreated control, ^†^*P*<0.05 when compared with the ISO-treated control.

**Figure 5:**
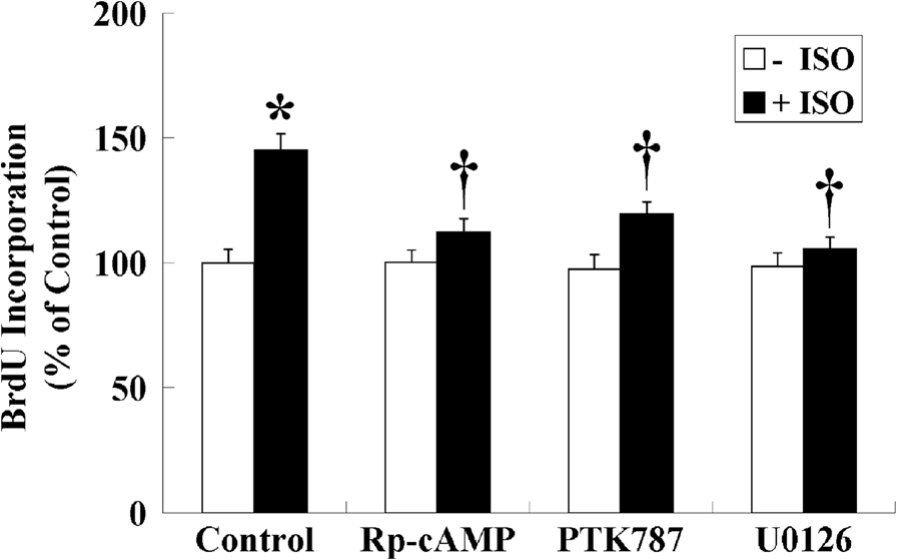
**Role of cAMP production and VEGFR-2 and ERK activation on ISO-induced cell proliferation.** HemECs were pre-treated with the cAMP antagonist, Rp-cAMP (10 μM), the VEGFR-2 inhibitor, PTK787 (10 μM), or the MAPK kinase inhibitor, U0126 (1 μM), for 1 h, incubated with 1 μM ISO for 12 h, and pulsed with 1:2,000 BrdU for 18 h. The results are shown as the mean ± SD of triplicate assays from one of three identical experiments. * *P*<0.05 when compared with the ISO-untreated control, ^†^*P*<0.05 when compared with the ISO-treated control.

In the classic model of β-adrenergic signaling, receptor activation results in the dissociation of the heterotrimeric G-protein, and the Gα_s_ subunit stimulates adenylyl cyclase to produce cAMP and activate the downstream protein kinase A (PKA)-mediated signaling pathway [33]. To determine whether activation of the β-ARs in HemECs resulted in the production of cAMP, intracellular levels of cAMP were measured in the presence or absence of ISO. Treatment with 1 μM ISO for 5 min produced a significant increase in cAMP production in HemECs; cAMP levels were increased by nearly 3.4-fold relative to the control. However, the increased cAMP levels induced by ISO were significantly reduced by pre-treatment with the β-AR antagonists (Figure 4). In addition, pre-treatment of cells with the cAMP antagonist, Rp-cAMP, prevented the ISO-induced proliferation of cell (Figure 5).

### PTK787 and U0126 abolished the stimulatory effect of ISO on cell proliferation

VEGFR-2 is the most biologically important receptor for VEGF-A in tumors. It regulates endothelial cell migration, proliferation and survival. Following the binding of VEGF-A, VEGFR-2 dimerizes and autophosphorylates the tyrosine residues in its cytoplasmic domain [34,35]. Tyr1175 is one of the major autophosphorylation sites in VEGFR-2, and phosphorylation of Tyr1175 mediates the activation of the MAP kinase ERK, which is essential in regulating endothelial cell proliferation [36].

To verify whether VEGFR-2 and ERK were involved in ISO-induced cell proliferation, HemECs were pre-treated with pharmacological inhibitors of VEGFR-2 (PTK787) and ERK (U0126) and were stimulated with 1 μM ISO. The results showed that pre-treatment with PTK787 significantly inhibited the ISO-induced cell proliferation of HemECs, and U0126 caused a greater decrease in the ISO-induced cell proliferation (Figure 5).

### The ISO-induced phosphorylation of ERK was abolished by β-AR antagonists and PTK787

**Figure 6:**
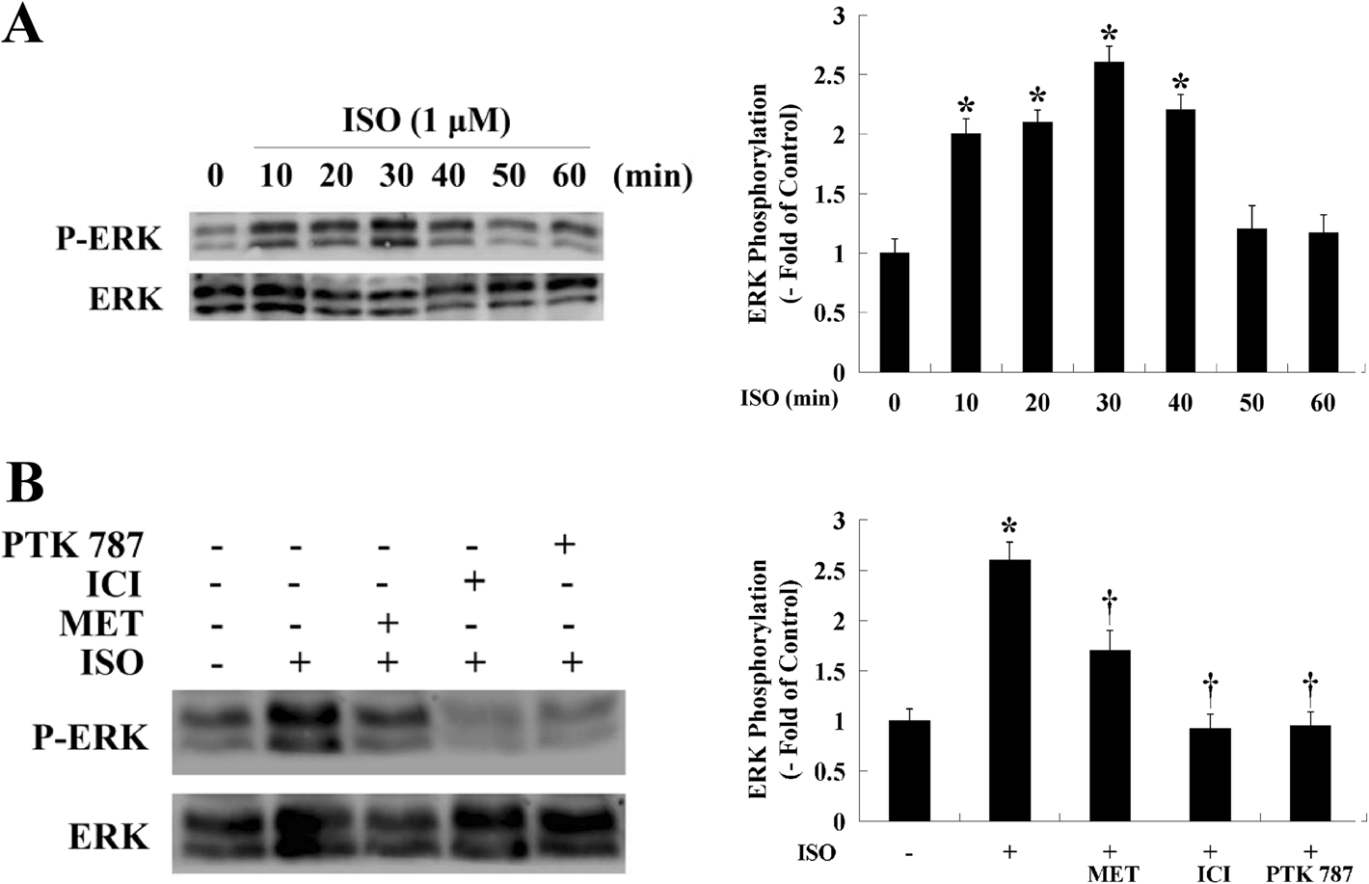
**Effect of β-AR stimulation on ERK activation in HemECs. A**, HemECs were treated with ISO for various times (0-60 min), and ERK phosphorylation was detected by Western blotting. ERK phosphorylation peaked 30 min after ISO treatment. **B**, HemECs were pre-treated with MET (10 μM), ICI (10 μM) or PTK787 (10 μM) for 1 h and incubated with ISO (1 μM) for 30 min. The phosphorylation status of ERK was then detected. Total ERK was used as an internal control. The experiments were repeated three times, and similar results were obtained. * *P*<0.05 when compared with the ISO-untreated control, ^†^*P*<0.05 when compared with the ISO-treated control.

Because the ISO-induced proliferation of HemECs was reduced by pre-treatment with an ERK inhibitor, ERK may be involved in the signal transduction pathway that is activated by ISO. To investigate this hypothesis, changes in the phosphorylation status of ERK were determined. Equal amounts of cell lysates were used to detect activated ERK using anti-phospho-ERK antibodies. The results showed that treating cells with ISO significantly increased ERK phosphorylation, which reached a maximum 30 min after ISO treatment (Figure 6A). Pre-treating the HemECs with MET or ICI significantly decreased ERK phosphorylation, indicating that the ISO-induced cell proliferation of HemECs was dependent on the activity of ERK. Next, we assessed whether ISO-mediated ERK activation was dependent on VEGFR-2 activity. In the presence of PTK787, ISO-mediated ERK activation was inhibited (Figure 6B).

### ISO increased VEGF-A expression and VEGFR-2 activation, both of which were inhibited by ICI

**Figure 7:**
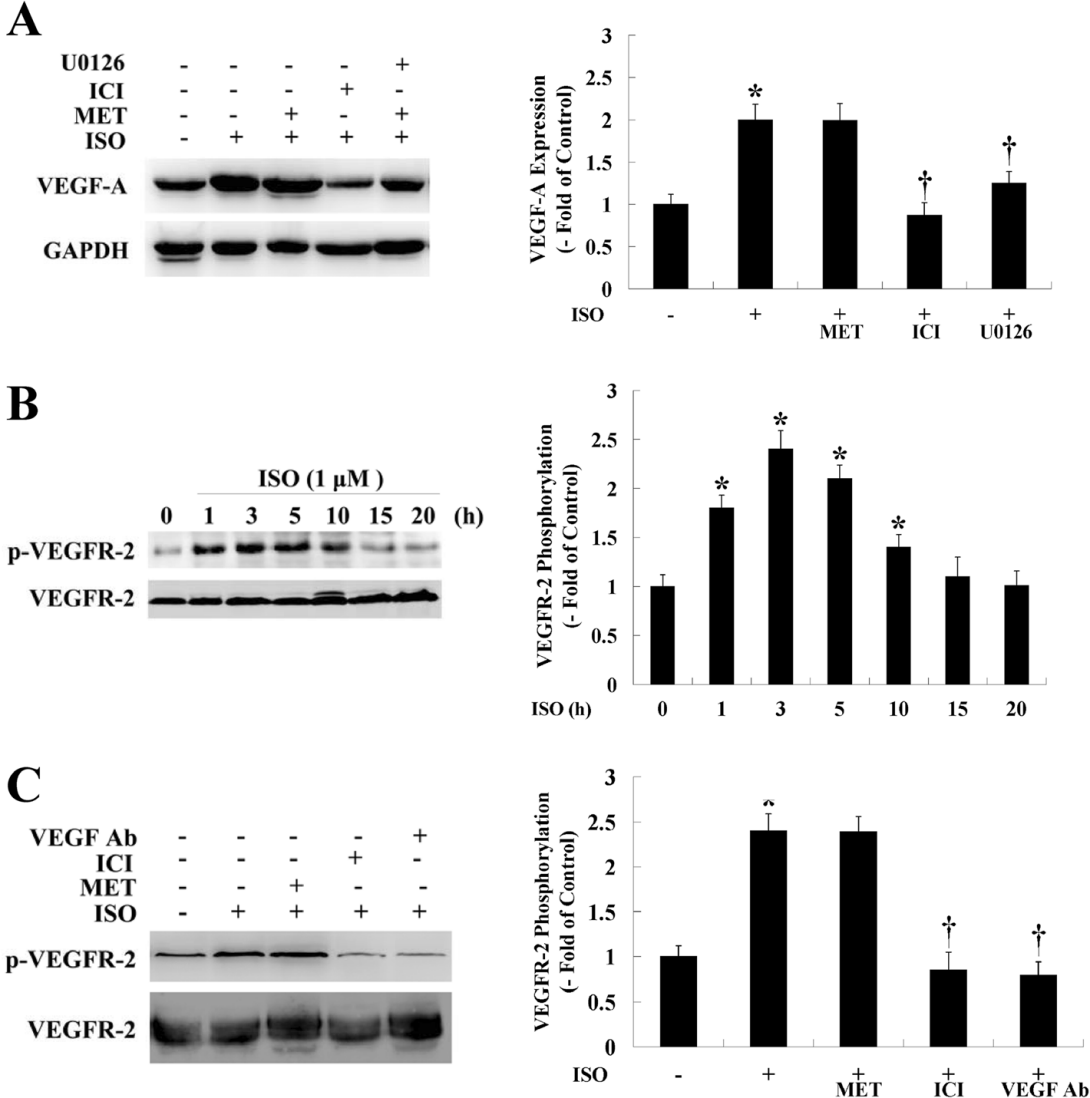
**Effect of β-AR stimulation on VEGF-A expression and VEGFR-2 activation in HemECs. A**, HemECs were pre-treated with MET (10 μM), ICI (10 μM) or U0126 (1 μM) for 1 h and incubated with ISO (1 μM) for 12 h. The expression of VEGF was then measured. GAPDH was used as an internal control. **B**, HemECs were treated with ISO for various times (0-20 h), and the phosphorylation level of VEGFR-2 was detected by Western blot using a phospho-specific antibody against the VEGFR-2 Tyr1175 residue. The ISO-induced phosphorylation of VEGFR-2 at Tyr1175 followed a bell-shaped curve starting at 1 h, reached a peak at 3 h and declined to basal levels after 15 h of treatment. **C**, HemECs were pre-treated with MET (10 μM), ICI (10 μM) or 2 μg/ml of a VEGF neutralizing antibody (VEGF Ab) for 1 h before incubating the cells with ISO (1 μM) for 3 h. The phosphorylation status of VEGFR-2 was then detected. **B** and **C** denote the mean ± SD of three experiments for each condition and was determined by the densitometry values that were determined relative to the total amounts of VEGFR-2. * *P*<0.05 when compared with the ISO-untreated control, ^†^*P*<0.05 when compared with the ISO-treated control.

We next examined whether ISO had an effect on VEGF-A expression and phosphorylation of VEGFR-2 at Tyr1175. The results showed that treating HemECs with ISO significantly increased VEGF-A expression. In contrast, pre-treatment of cells with ICI or U0126 significantly suppressed VEGF-A expression (Figure 7A). VEGFR-2 phosphorylation peaked 3 h after ISO treatment (Figure 7B). Pre-treating HemECs with ICI or a VEGF neutralizing antibody significantly abolished VEGFR-2 phosphorylation. However, MET had no effect on ISO-induced VEGF-A expression or VEGFR-2 phosphorylation. (Figure 7A, C).

## Discussion

β-ARs have been reported to participate in the promotion and progression of different neoplasms, including various types of adenocarcinomas and squamous-cell carcinomas. In those studies, cancer cell growth was stimulated either by the non-selective adrenergic agonists or more effectively by the β-selective agonists [26,31,37-39]. The authors suggested that the tumor cells might secrete low levels of catecholamines to self-stimulate their growth via the β-ARs [40,41]. It is known that agonist and antagonist of β-ARs act antithetic via same intracellular pathways. Recently, β-AR antagonists (e.g., propranolol) have been found to provide therapeutic leverage in the context of breast cancer [42-44], melanoma [45,46] and IH [13-21].

HemECs exhibit an X-chromosome inactivation pattern of clonality, and show upregulation of some markers and downregulation of others. This expression pattern is stably maintained in cultured HemECs and differs from that of other endothelial cells [8,47,48]. In the present study, we demonstrated that the β_1_- and β_2_-ARs were expressed in HemECs. Activation of the β-ARs resulted in an increased concentration of intracellular cAMP and enhanced cell proliferation, two processes that could be reversed by treatment with β_1_- or β_2_-AR antagonists. Interestingly, β-AR stimulation has recently been demonstrated to be a major factor that contributes to the initiation of IH by Mayer et al. [49], who found that intrauterine exposure to β_2_-sympathomimetic hexoprenaline can increase the occurrence of IH in preterm infants. In addition, the β_2_-AR antagonist but not the β_1_-AR antagonist completely abolished ISO-induced cell proliferation, suggesting that the mitogenic effect of ISO predominately occurred through the β_2_-AR. This finding is in agreement with a previous report that showed that the stimulatory effect of ISO on aorta endothelial cells was preferentially mediated by the β_2_-AR [25]. However, it was reported that the current selective β_1_-blockers in use are not entirely β_1_ specific. In fact, MET partially inhibits β_2_-AR as well [50]. It is therefore possible that even limited β_2_-adrenergic inhibition by MET might be sufficient to inhibit cell proliferation.

Control of cell cycle progression in tumor cells may be an effective strategy for treating tumors. The current findings clearly showed that the β-AR antagonists arrested ISO-treated cells at the G_0_/G_1_ phase of the cell cycle, suggesting that the β-AR antagonists inhibited cell proliferation via interactions with cell cycle regulators. Indeed, cyclin D1, CDK-4, CDK-6 and phospho-Rb have been reported to control the vascular endothelial cell proliferation during pathogenic neovascularization [51]. We investigated whether the expression of these established cell cycle regulators was controlled by the β-ARs in HemECs. Our results showed that treatment of HemECs with ISO resulted in a moderate to strong increase in the protein levels of cyclin D1, CDK-4, CDK-6 and phospho-Rb, but these high levels of expression were reversed by pre-treatment with either the β_1_- or β_2_-AR antagonist. The mechanism responsible for these changes remains unknown and merits further investigation.

ERK proteins are reversibly phosphorylated by a variety of protein kinases and upstream signaling molecules as a result of the activation of receptor tyrosine kinases and G-protein-coupled receptors. The β-ARs promoted vascular endothelial cell ERK activation by at least two mechanisms. First, stimulation of endothelial β-ARs directly activated ERK signaling cascades, and second, β-AR stimulation induced the release of VEGF-A, which can also activate ERK [25]. In the present study, ERK inhibition prevented HemEC proliferation, demonstrating that this kinase is critical for β-AR-mediated cell mitogenesis and proliferation. Moreover, ISO significantly induced ERK activation, and this effect was abolished by either the β_1_ or β_2_-AR antagonist.

Exposure to a chronic stressor promoted in vivo angiogenesis and production of VEGF. This effect was eliminated by silencing tumor cell β-AR expression, implicating tumor cell β-AR expression and signaling as an important facilitator of stress-induced tumor angiogenesis in vivo [39]. In vitro studies using tumor cell lines suggest that catecholamines can promote tumor progression by a β-AR-driven proangiogenic pathway. This stimulation of VEGF expression by β-adrenergic signaling is proportional to β-AR expression, dose-dependent and inhibited by β-AR antagonists [37,52]. There is evidence that expression of VEGF in endothelial cells may also be controlled by adrenergic stimulation; as demonstrated in different in vitro and in vivo models, β-AR agonists, including epinephrine, norepinephrine and ISO, can induce the expression of VEGF [53-55]. Conversely, β-AR antagonists (e.g., propranolol) lead to a reduced expression of VEGF and inhibit cell proliferation and angiogenesis [29,56]. In the present study, ISO increased the expression level of VEGF-A in HemECs in a β-AR- and ERK-dependent manner. These findings are consistent with previous studies in which β-AR stimulation resulted in the over-expression of VEGF-A through the β-AR and ERK signaling cascade [25,31,57].

We also found that the ISO-stimulated activation of ERK and subsequent proliferation of HemECs required VEGFR-2 activity. Studies have shown that cultured HemECs share a phenotype of constitutively active VEGFR-2 signaling, which might render the cells more sensitive to autocrine or paracrine stimulation of VEGF-A [9]. The VEGFR-2 intracellular signaling pathway in HemECs was not fully explored, but results from the in vitro VEGF-A stimulation of different types of endothelial cells indicated that VEGFR-2 signaling is dependent on the downstream effects of ERK [29,30,34]. Although activation of VEGFR-2 and β-ARs has been implicated in the promotion of cell proliferation, the connection between these two receptor systems is poorly understood. Here, we provide the first evidence that the VEGFR-2-mediated phosphorylation of ERK is upregulated upon β-AR activation to mediate proliferation of HemECs. These findings, together with the observation that the ISO-induced phosphorylation of VEGFR-2 could be inhibited by ICI, demonstrate that the transactivation of VEGFR-2 may act as an effector pathway to mediate the mitogenic effects of the β-ARs.

In conclusion, we demonstrated that activation of the β-ARs resulted in increased HemEC proliferation and upregulation of the ERK signaling cascade. VEGFR-2-mediated ERK signaling was also upregulated upon β-AR activation to mediate proliferation of HemECs. These findings not only provide a pharmacological basis for the therapeutic use of β-AR antagonists in the treatment of IH but also unveil a functional connection between the β-ARs and VEGFR-2 in HemECs.

## Competing interests

The authors declare that they have no competing interest.

## Authors’ contributions

YJ and SYC contributed to the design of the study, planned and performed the experiments, interpreted the statistical analysis, and drafted the manuscript. KL and XMX contributed to the conception and design of the study and revised the manuscript. SZ revised the manuscript. TX performed the experiments and conducted the statistical analysis. All of the authors read and approved the final manuscript.

## References

[B1] Drolet BA Esterly NB Frieden IJ Hemangiomas in children N Engl J Med 1999 341 3 173 181 10.1056/NEJM199907153410307 10403856

[B2] Mulliken JB Fishman SJ Burrows PE Vascular anomalies Curr Probl Surg 2000 37 8 517 584 10.1016/S0011-3840(00)80013-1 10955029

[B3] Drolet BA Swanson EA Frieden IJ Infantile hemangiomas: an emerging health issue linked to an increased rate of low birth weight infants J Pediatr 2008 153 5 712 715 711-715 10.1016/j.jpeds.2008.05.043 18940356

[B4] Frieden IJ Haggstrom AN Drolet BA Mancini AJ Friedlander SF Boon L Chamlin SL Baselga E Garzon MC Nopper AJ Infantile hemangiomas: current knowledge, future directions. Proceedings of a research workshop on infantile hemangiomas, April 7-9, 2005, Bethesda, Maryland, USA Pediatr Dermatol 2005 22 5 383 406 10.1111/j.1525-1470.2005.00102.x 16190987

[B5] Takahashi K Mulliken JB Kozakewich HP Rogers RA Folkman J Ezekowitz RA Cellular markers that distinguish the phases of hemangioma during infancy and childhood J Clin Invest 1994 93 6 2357 2364 10.1172/JCI117241 7911127 PMC294441

[B6] Tan ST Velickovic M Ruger BM Davis PF Cellular and extracellular markers of hemangioma Plast Reconstr Surg 2000 106 3 529 538 10987458 10.1097/00006534-200009030-00001

[B7] Greenberger S Boscolo E Adini I Mulliken JB Bischoff J Corticosteroid suppression of VEGF-A in infantile hemangioma-derived stem cells N Engl J Med 2010 362 11 1005 1013 10.1056/NEJMoa0903036 20237346 PMC2845924

[B8] Boye E Yu Y Paranya G Mulliken JB Olsen BR Bischoff J Clonality and altered behavior of endothelial cells from hemangiomas J Clin Invest 2001 107 6 745 752 10.1172/JCI11432 11254674 PMC208946

[B9] Jinnin M Medici D Park L Limaye N Liu Y Boscolo E Bischoff J Vikkula M Boye E Olsen BR Suppressed NFAT-dependent VEGFR1 expression and constitutive VEGFR2 signaling in infantile hemangioma Nat Med 2008 14 11 1236 1246 10.1038/nm.1877 18931684 PMC2593632

[B10] George ME Sharma V Jacobson J Simon S Nopper AJ Adverse effects of systemic glucocorticosteroid therapy in infants with hemangiomas Arch Dermatol 2004 140 8 963 969 10.1001/archderm.140.8.963 15313812

[B11] Goyal R Watts P Lane CM Beck L Gregory JW Adrenal suppression and failure to thrive after steroid injections for periocular hemangioma Ophthalmology 2004 111 2 389 395 10.1016/S0161-6420(03)00833-9 15019396

[B12] Chang LC Haggstrom AN Drolet BA Baselga E Chamlin SL Garzon MC Horii KA Lucky AW Mancini AJ Metry DW Growth characteristics of infantile hemangiomas: implications for management Pediatrics 2008 122 2 360 367 10.1542/peds.2007-2767 18676554

[B13] Leaute-Labreze C Dumas DLRE Hubiche T Boralevi F Thambo JB Taieb A Propranolol for severe hemangiomas of infancy N Engl J Med 2008 358 24 2649 2651 10.1056/NEJMc0708819 18550886

[B14] Sans V de la Roque ED Berge J Grenier N Boralevi F Mazereeuw-Hautier J Lipsker D Dupuis E Ezzedine K Vergnes P Propranolol for severe infantile hemangiomas: follow-up report Pediatrics 2009 124 3 e423 e431 10.1542/peds.2008-3458 19706583

[B15] Leboulanger N Fayoux P Teissier N Cox A Van Den Abbeele T Carrabin L Couloigner V Nicollas R Triglia JM Ayari S Propranolol in the therapeutic strategy of infantile laryngotracheal hemangioma: a preliminary retrospective study of French experience Int J Pediatr Otorhinolaryngol 2010 74 11 1254 1257 10.1016/j.ijporl.2010.07.025 20800295

[B16] Manunza F Syed S Laguda B Linward J Kennedy H Gholam K Glover M Giardini A Harper JI Propranolol for complicated infantile haemangiomas: a case series of 30 infants Br J Dermatol 2010 162 2 466 468 10.1111/j.1365-2133.2009.09597.x 20055816

[B17] Truong MT Chang KW Berk DR Heerema-McKenney A Bruckner AL Propranolol for the treatment of a life-threatening subglottic and mediastinal infantile hemangioma J Pediatr 2010 156 2 335 338 10.1016/j.jpeds.2009.10.010 20105647

[B18] Fuchsmann C Quintal MC Giguere C Ayari-Khalfallah S Guibaud L Powell J McCone C Froehlich P Propranolol as first-line treatment of head and neck hemangiomas Arch Otolaryngol Head Neck Surg 2011 137 5 471 478 10.1001/archoto.2011.55 21576558

[B19] Hogeling M Adams S Wargon O A randomized controlled trial of propranolol for infantile hemangiomas Pediatrics 2011 128 2 e259 e266 10.1542/peds.2010-0029 21788220

[B20] Schupp CJ Kleber JB Gunther P Holland-Cunz S Propranolol therapy in 55 infants with infantile hemangioma: dosage, duration, adverse effects, and outcome Pediatr Dermatol 2011 28 6 640 644 10.1111/j.1525-1470.2011.01569.x 21995836

[B21] Hong E Fischer G Propranolol for recalcitrant ulcerated hemangioma of infancy Pediatr Dermatol 2012 29 1 64 67 10.1111/j.1525-1470.2011.01547.x 21854419

[B22] Anitole-Misleh KG Brown KM Developmental regulation of catecholamine levels during sea urchin embryo morphogenesis Comp Biochem Physiol A Mol Integr Physiol 2004 137 1 39 50 10.1016/j.cbpb.2003.09.001 14720589

[B23] Kim MO Na SI Lee MY Heo JS Han HJ Epinephrine increases DNA synthesis via ERK1/2 s through cAMP, Ca(2+)/PKC, and PI3K/Akt signaling pathways in mouse embryonic stem cells J Cell Biochem 2008 104 4 1407 1420 10.1002/jcb.21716 18275042

[B24] Herlenius E Lagercrantz H Neurotransmitters and neuromodulators during early human development Early Hum Dev 2001 65 1 21 37 10.1016/S0378-3782(01)00189-X 11520626

[B25] Iaccarino G Ciccarelli M Sorriento D Galasso G Campanile A Santulli G Cipolletta E Cerullo V Cimini V Altobelli GG Ischemic neoangiogenesis enhanced by beta2-adrenergic receptor overexpression: a novel role for the endothelial adrenergic system Circ Res 2005 97 11 1182 1189 10.1161/01.RES.0000191541.06788.bb 16239589

[B26] Lutgendorf SK Cole S Costanzo E Bradley S Coffin J Jabbari S Rainwater K Ritchie JM Yang M Sood AK Stress-related mediators stimulate vascular endothelial growth factor secretion by two ovarian cancer cell lines Clin Cancer Res 2003 9 12 4514 4521 14555525

[B27] Lai KB Sanderson JE Yu CM The regulatory effect of norepinephrine on connective tissue growth factor (CTGF) and vascular endothelial growth factor (VEGF) expression in cultured cardiac fibroblasts Int J Cardiol 2011 In press 10.1016/j.ijcard.2011.06.003 21704393

[B28] Schuller HM Beta-adrenergic signaling, a novel target for cancer therapy? Oncotarget 2010 1 7 466 469 21317444 10.18632/oncotarget.182 PMC3248132

[B29] Lamy S Lachambre MP Lord-Dufour S Beliveau R Propranolol suppresses angiogenesis in vitro: inhibition of proliferation, migration, and differentiation of endothelial cells Vascul Pharmacol 2010 53 5–6 200 208 20732454 10.1016/j.vph.2010.08.002

[B30] Takahashi T Ueno H Shibuya M VEGF activates protein kinase C-dependent, but Ras-independent Raf-MEK-MAP kinase pathway for DNA synthesis in primary endothelial cells Oncogene 1999 18 13 2221 2230 10.1038/sj.onc.1202527 10327068

[B31] Liu X Wu WK Yu L Sung JJ Srivastava G Zhang ST Cho CH Epinephrine stimulates esophageal squamous-cell carcinoma cell proliferation via beta-adrenoceptor-dependent transactivation of extracellular signal-regulated kinase/cyclooxygenase-2 pathway J Cell Biochem 2008 105 1 53 60 10.1002/jcb.21802 18452159

[B32] Guo Y Yang K Harwalkar J Nye JM Mason DR Garrett MD Hitomi M Stacey DW Phosphorylation of cyclin D1 at Thr 286 during S phase leads to its proteasomal degradation and allows efficient DNA synthesis Oncogene 2005 24 16 2599 2612 10.1038/sj.onc.1208326 15735756

[B33] Rybin VO Xu X Lisanti MP Steinberg SF Differential targeting of beta -adrenergic receptor subtypes and adenylyl cyclase to cardiomyocyte caveolae. A mechanism to functionally regulate the cAMP signaling pathway J Biol Chem 2000 275 52 41447 41457 10.1074/jbc.M006951200 11006286

[B34] Cho CH Lee CS Chang M Jang IH Kim SJ Hwang I Ryu SH Lee CO Koh GY Localization of VEGFR-2 and PLD2 in endothelial caveolae is involved in VEGF-induced phosphorylation of MEK and ERK Am J Physiol Heart Circ Physiol 2004 286 5 H1881 H1888 10.1152/ajpheart.00786.2003 14704231

[B35] Kendall RL Rutledge RZ Mao X Tebben AJ Hungate RW Thomas KA Vascular endothelial growth factor receptor KDR tyrosine kinase activity is increased by autophosphorylation of two activation loop tyrosine residues J Biol Chem 1999 274 10 6453 6460 10.1074/jbc.274.10.6453 10037737

[B36] Takahashi T Yamaguchi S Chida K Shibuya M A single autophosphorylation site on KDR/Flk-1 is essential for VEGF-A-dependent activation of PLC-gamma and DNA synthesis in vascular endothelial cells EMBO J 2001 20 11 2768 2778 10.1093/emboj/20.11.2768 11387210 PMC125481

[B37] Park SY Kang JH Jeong KJ Lee J Han JW Choi WS Kim YK Kang J Park CG Lee HY Norepinephrine induces VEGF expression and angiogenesis by a hypoxia-inducible factor-1alpha protein-dependent mechanism Int J Cancer 2011 128 10 2306 2316 10.1002/ijc.25589 20715173

[B38] Yang EV Sood AK Chen M Li Y Eubank TD Marsh CB Jewell S Flavahan NA Morrison C Yeh PE Norepinephrine up-regulates the expression of vascular endothelial growth factor, matrix metalloproteinase (MMP)-2, and MMP-9 in nasopharyngeal carcinoma tumor cells Cancer Res 2006 66 21 10357 10364 10.1158/0008-5472.CAN-06-2496 17079456

[B39] Sloan EK Priceman SJ Cox BF Yu S Pimentel MA Tangkanangnukul V Arevalo JM Morizono K Karanikolas BD Wu L The sympathetic nervous system induces a metastatic switch in primary breast cancer Cancer Res 2010 70 18 7042 7052 10.1158/0008-5472.CAN-10-0522 20823155 PMC2940980

[B40] Wu WK Wong HP Luo SW Chan K Huang FY Hui MK Lam EK Shin VY Ye YN Yang YH 4-(Methylnitrosamino)-1-(3-pyridyl)-1-butanone from cigarette smoke stimulates colon cancer growth via beta-adrenoceptors Cancer Res 2005 65 12 5272 5277 10.1158/0008-5472.CAN-05-0205 15958573

[B41] Wong HP Yu L Lam EK Tai EK Wu WK Cho CH Nicotine promotes cell proliferation via alpha7-nicotinic acetylcholine receptor and catecholamine-synthesizing enzymes-mediated pathway in human colon adenocarcinoma HT-29 cells Toxicol Appl Pharmacol 2007 221 3 261 267 10.1016/j.taap.2007.04.002 17498763

[B42] Barron TI Connolly RM Sharp L Bennett K Visvanathan K Beta blockers and breast cancer mortality: a population- based study J Clin Oncol 2011 29 19 2635 2644 10.1200/JCO.2010.33.5422 21632503

[B43] Powe DG Voss MJ Zanker KS Habashy HO Green AR Ellis IO Entschladen F Beta-blocker drug therapy reduces secondary cancer formation in breast cancer and improves cancer specific survival Oncotarget 2010 1 7 628 638 21317458 10.18632/oncotarget.197 PMC3248123

[B44] Melhem-Bertrandt A Chavez-Macgregor M Lei X Brown EN Lee RT Meric-Bernstam F Sood AK Conzen SD Hortobagyi GN Gonzalez-Angulo AM Beta-blocker use is associated with improved relapse-free survival in patients with triple-negative breast cancer J Clin Oncol 2011 29 19 2645 2652 10.1200/JCO.2010.33.4441 21632501 PMC3139371

[B45] De Giorgi V Grazzini M Gandini S Benemei S Lotti T Marchionni N Geppetti P Treatment with beta-blockers and reduced disease progression in patients with thick melanoma Arch Intern Med 2011 171 8 779 781 10.1001/archinternmed.2011.131 21518948

[B46] Lemeshow S Sorensen HT Phillips G Yang EV Antonsen S Riis AH Lesinski GB Jackson R Glaser R beta-Blockers and survival among Danish patients with malignant melanoma: a population-based cohort study Cancer Epidemiol Biomarkers Prev 2011 20 10 2273 2279 10.1158/1055-9965.EPI-11-0249 21933972 PMC3652234

[B47] Dosanjh A Chang J Bresnick S Zhou L Reinisch J Longaker M Karasek M In vitro characteristics of neonatal hemangioma endothelial cells: similarities and differences between normal neonatal and fetal endothelial cells J Cutan Pathol 2000 27 9 441 450 10.1034/j.1600-0560.2000.027009441.x 11028814

[B48] Yu Y Varughese J Brown LF Mulliken JB Bischoff J Increased Tie2 expression, enhanced response to angiopoietin-1, and dysregulated angiopoietin-2 expression in hemangioma-derived endothelial cells Am J Pathol 2001 159 6 2271 2280 10.1016/S0002-9440(10)63077-5 11733376 PMC1850579

[B49] Mayer M Minichmayr A Klement F Hroncek K Wertaschnigg D Arzt W Wiesinger-Eidenberger G Lechner E Tocolysis with the beta-2-sympathomimetic hexoprenaline increases occurrence of infantile haemangioma in preterm infants Arch Dis Child Fetal Neonatal Ed 2012 In press 10.1136/archdischild-2011-301030 22611112

[B50] Smith C Teitler M Beta-blocker selectivity at cloned human beta 1- and beta 2-adrenergic receptors Cardiovasc Drugs Ther 1999 13 2 123 126 10.1023/A:1007784109255 10372227

[B51] Alhaja E Adan J Pagan R Mitjans F Cascallo M Rodriguez M Noe V Ciudad CJ Mazo A Vilaro S Anti-migratory and anti-angiogenic effect of p16: a novel localization at membrane ruffles and lamellipodia in endothelial cells Angiogenesis 2004 7 4 323 333 10.1007/s10456-005-0368-9 15886876

[B52] Ciccarelli M Sorriento D Cipolletta E Santulli G Fusco A Zhou RH Eckhart AD Peppel K Koch WJ Trimarco B Impaired neoangiogenesis in beta(2)-adrenoceptor gene-deficient mice: restoration by intravascular human beta(2)-adrenoceptor gene transfer and role of NFkappaB and CREB transcription factors Br J Pharmacol 2011 162 3 712 721 10.1111/j.1476-5381.2010.01078.x 20958287 PMC3041259

[B53] Steinle JJ Cappocia FJ Jiang Y Beta-adrenergic receptor regulation of growth factor protein levels in human choroidal endothelial cells Growth Factors 2008 26 6 325 330 10.1080/08977190802442070 19021032

[B54] Seya Y Fukuda T Isobe K Kawakami Y Takekoshi K Effect of norepinephrine on RhoA, MAP kinase, proliferation and VEGF expression in human umbilical vein endothelial cells Eur J Pharmacol 2006 553 1–3 54 60 17070516 10.1016/j.ejphar.2006.09.048

[B55] Tilan J Kitlinska J Sympathetic neurotransmitters and tumor angiogenesis-link between stress and cancer progression J Oncol 2010 2010 539706 20508839 10.1155/2010/539706 PMC2874925

[B56] Annabi B Lachambre MP Plouffe K Moumdjian R Beliveau R Propranolol adrenergic blockade inhibits human brain endothelial cells tubulogenesis and matrix metalloproteinase-9 secretion Pharmacol Res 2009 60 5 438 445 10.1016/j.phrs.2009.05.005 19467330

[B57] Schuller HM Al-Wadei HA Ullah MF Plummer HR Regulation of pancreatic cancer by neuropsychological stress responses: a novel target for intervention Carcinogenesis 2012 33 1 191 196 10.1093/carcin/bgr251 22072614 PMC3276326

